# Patients with follicular lymphoma should be treated according to baseline PET-CT findings with targeted re-biopsy

**DOI:** 10.1007/s00277-025-06609-2

**Published:** 2025-10-06

**Authors:** Anni Nyyssönen, Outi Kuittinen, Tero Vuolio, Elias Vaattovaara, Aino Rajamäki, Taina Turpeenniemi-Hujanen, Marc Sorigue, Hanne Kuitunen, Milla E.L. Kuusisto

**Affiliations:** 1 Department of Medicine, University of Oulu, Oulu, Finland; 2 Faculty of Health Medicine, Institute of Clinical Medicine, University of Eastern Finland, Kuopio, Finland; 3 Department of Clinical Oncology, Kuopio University Hospital, Kuopio, Finland; 4 Department of Radiology, Oulu University Hospital, Oulu, Finland; 5 Department of Oncology, Tays Cancer Center, Tampere University Hospital, Tampere, Finland; 6 Research Unit of Translational Medicine, University of Oulu, Oulu, Finland; 7 Cancer Center, Oulu University Hospital, Oulu, Finland; 8 Medical Department, Trialing Health, Barcelona, Spain; 9 Department of Internal Medicine, Länsi-Pohja Central Hospital, Kemi, Finland; 10 Medical Research Center, Oulu University Hospital, Oulu, Finland; 11 Biomedicine and Internal Medicine Research Unit, University of Oulu, Oulu, Finland

**Keywords:** Follicular lymphoma, PET-CT, POD24, Re-biopsy, Transformation

## Abstract

Although the increasing use of PET-CT has enabled improvements in staging and confirmation of a suspected histological transformation of follicular lymphoma (FL), the disease is still characterised by varied courses and outcomes. Our aim was to determine, whether diagnostic PET-CT could be effective in preventing early progression, particularly disease progression within 24 months of started therapy (POD24). Patient data of 177 grade 1-3a FL patients treated in Oulu University Hospital between years 2000 and 2020 was retrospectively reviewed. Staging of 59 patients included PET-CT before first-line treatment, when excluding two patients who were found to be primary transformed based on their PET-CT. 25 (42.4%) of the 59 patients were also re-biopsied based on the staging results. The control group consisted of 118 non-PET-CT staged patients who received systemic therapy for their disease. The use of PET-CT at the time of the diagnosis was determined by clinician based on the clinical course of the individual patient. In the PET-CT staged group four transformations were noted during follow-up and six cases of POD24 occurred. In comparison, fifteen transformations (*p* = 0.306) and 18 POD24 (*p* = 0.486) occurred in the reference group. A high SUVmax was indicative of worse outcomes (*p* = 0.016) but survival was not improved in the re-biopsied subgroup. Diagnostic PET-CT enhanced disease course as time to progression was superior in the group of PET-CT staged patients (*p* = 0.038). Our results suggest that PET-CT is a valuable diagnostic tool at baseline which may help to identify patients at risk for POD24 leading to better survival.

## Introduction

While grade 1-3a follicular lymphoma (FL), also known as classic FL according to WHO-HAEM5 [1], is characterized as an indolent non-Hodgkin lymphoma (NHL) with an improved survival after the introduction of immunochemotherapy, the ~ 20% of patients with FL who still experience progression of disease within 24 months of first-line therapy (POD24) have unfavourable outcome [2, 3]. Besides the risk of early disease progression, a histological transformation of FL to a more aggressive NHL may occur in the course of FL. Similarities between the predictable factors of POD24 and disease transformation have been noted while both additionally possess a clear risk for increased lymphoma related mortality [4].

It may be possible for FL to transform in one site while maintaining an indolent histology elsewhere with no suspected clinical evidence [5]. However, in cases of FL transformation, 18 F-fluorodeoxyglucose positron emission tomography-computed tomography (PET-CT) imaging has been shown to present higher maximum standardized uptake values (SUVmax) on transformed sites [6]. Therefore, PET-CT scanning has become the standard in addition to improve staging accuracy, also to identify an optimal biopsy site in cases of clinically suspected malignant transformation [7].

The aim of the study is a comparison of the clinical consequences of a staging procedure with or without PET-CT for FL staging. We aimed to assess whether a routine PET-CT scan at diagnosis, used also to guide a re-biopsy from the area with the highest SUVmax value, can be used to prevent POD24 or detect a histological transformation. There is a suspicion that patients at the risk of POD24 have higher SUVmax values than those whose disease is more indolent with its course. The evaluation was made by a retrospective comparison between two matched patient cohorts staged with or without PET-CT, treated in a single institution.

## Materials and methods

**Fig. 1:**
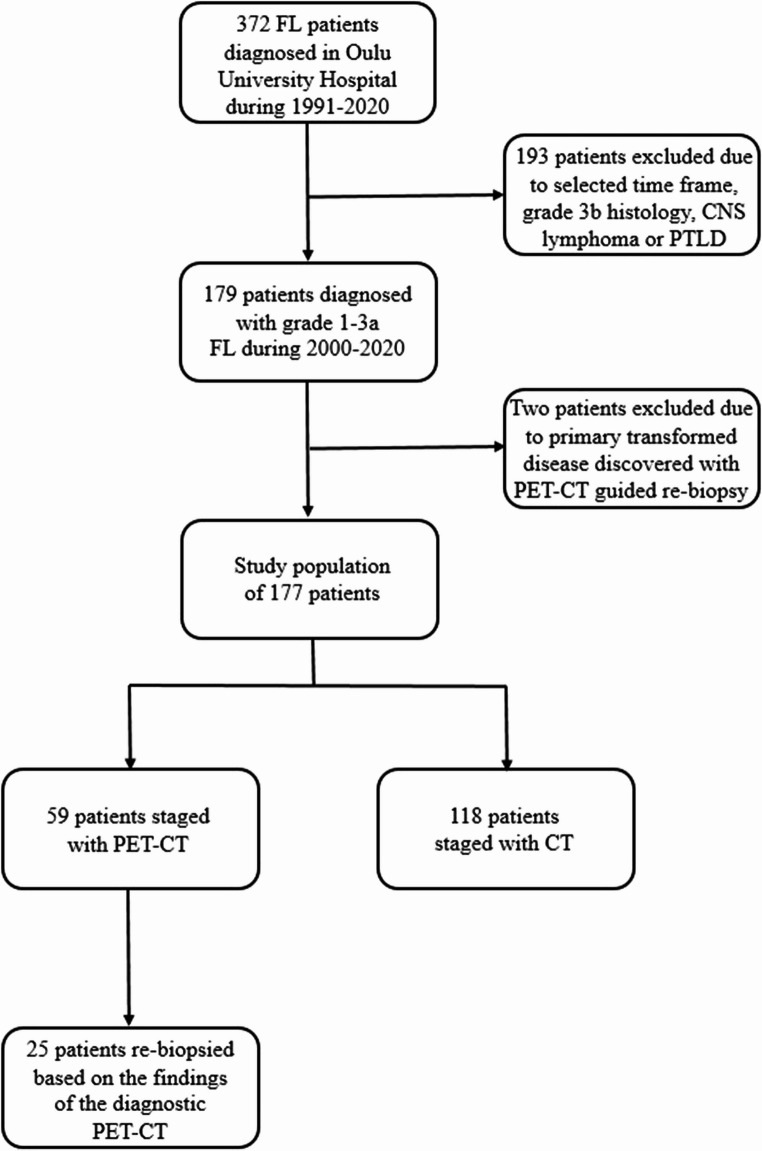
Flow chart of the study population of patients with newly diagnosed follicular lymphoma (FL). CNS, central nervous system; PTLD, post-transplant lymphoproliferative disorder

The patient material consisted of two different cohorts of FL patients who were staged according to Lugano criteria [8] with or without PET-CT, upon adoption of a systematic use of PET-CT for staging. Individual patient data for this study was retrospectively obtained from 177 patients diagnosed with FL, who were treated in Oulu University Hospital. In the present study, all of the eligible patients had a biopsy-proven FL of grade 1 to 3a diagnosed and treated between years 2000 and 2020 (Fig. 1). From this group of FL patients, 59 had diagnostic PET-CT scanning performed at the time of primary diagnosis or at latest before the first-line therapy was initiated. The decision to carry out diagnostic PET-CT was determined by clinician based on the clinical course of the individual patient. The recommendation for imaging was carried out according to Lugano criteria [8] for FL staging and because of clinical suspicion of histological transformation. 118 patients were not PET-CT scanned at baseline but were treated for their disease and were used as a control group in the present study. The reason for not using PET-CT staging was the availability of PET-CT scan and current clinical practise. Of the fifty-nine patients in the PET-CT staged group, 25 had a PET-CT scan guided re-biopsy performed at the time of the diagnosis before the initiation of the treatment. Moreover, available reported SUVmax values of the 59 patients were divided into two groups, with 31 patients having SUVmax values lower than 10, and 27 patients with ten or higher. The threshold value of 10 was based on the available literature suggesting that a SUVmax over 10 may predict a more aggressive histology of lymphoma [9]. This was also the threshold for considering re-biopsy. The SUVmax value of one patient was not reported in patient data or the images available for re-evaluation. The present study was conducted following the principles of the Declaration of Helsinki and approved by the Ethics Committee of the Northern Ostrobothnia Hospital District, Oulu University Hospital.

IBM SPSS Statistics for Windows (version 29; IBM Corporation, Armonk, NY) was used to perform the statistical analysis. POD24 was defined as the progression of FL in 24 months after initiation of first-line systemic therapy. Time to progression (TTP) was defined from the time of diagnosis to the date of confirmed progression (primary refractory disease or relapsed disease) or death as a result of lymphoma, or to the last follow-up date, which ever came first. Overall survival (OS) was defined from the time of diagnosis to death of any cause or the last follow-up date, which ever came first. Disease-specific survival (DSS) was similarly calculated from the time of diagnosis to lymphoma-related death or last follow-up date. The Kaplan-Meier survival analysis with log-rank test was used to calculate the TTP, OS and DSS times. Nominal variables were tested with chi square and Fisher’s exact test. Uni- and multivariate analysis were carried out with ANOVA and Cox regression model. Continuous variables were evaluated by using Student’s *t*-test. In the statistical tests, the *p-value* < 0.05 was considered statistically significant.

## Results

The characteristics of all patients divided by PET-CT imaging status are presented in Table 1. The median age at diagnosis was 61 (range, 20–88) of all 177 patients. In PET-CT staged the median was 65 (29–88) and in the reference group 60 years (20–87) (*p* = 0.602). Median time of follow-up was 105 months (1-281), 57 (1-171) for the PET-CT staged group and 142 (2-281) for patients in the reference group (*p* < 0.001). Median time from diagnosis to start of the first-line treatment was one month (0–42) in the PET-CT staged patients. Altogether, 24 cases of POD24 occurred during the follow-up, six in PET-CT staged and 18 in reference group (*p* = 0.486), respectively. During the follow-up of PET-CT staged patients, there were 12 deaths, of which 25.0% were lymphoma-associated. Moreover, 42 deaths occurred in the reference group, and of those 38.1% were due to FL (*p* = 0.506).

**Table 1:** Patient characteristics

Variable	PET-CT staged patients (*n* = 59) *n* (%)	Reference group (*n* = 118) *n* (%)	*p*
Sex			0.524
Male	33 (55.9)	59 (50.0)	
Female	26 (44.1)	59 (50.0)	
Age at diagnosis			0.107
Under 60 years	20 (33.9)	56 (47.5)	
60 years or over	39 (66.1)	62 (52.5)	
Stage			0.741
1–2	20 (33.9)	44 (37.3)	
3–4	39 (66.1)	74 (62.7)	
Grade			0.056
1–2	47 (79.7)	107 (90.7)	
3a	12 (20.3)	11 (9.3)	
LDH level			1.00
Normal	35 (59.3)	70 (59.3)	
Elevated	22 (37.3)	44 (37.3)	
NA	2 (3.4)	4 (3.4)	
Haemoglobin level			0.855
≥ 120	49 (83.1)	101 (85.6)	
< 120	9 (15.2)	14 (11.9)	
NA	1 (1.7)	3 (2.5)	
ECOG			1.00
0–1	55 (93.2)	111 (94.1)	
Over 1	3 (5.1)	6 (5.1)	
NA	1 (1.7)	1 (0.8)	
B-symptoms			0.571
Yes	16 (27.1)	27 (22.9)	
No	42 (71.2)	90 (76.3)	
NA	1 (1.7)	1 (0.8)	
FLIPI			0.738
0	8 (13.5)	11 (9.3)	
1–2	25 (42.4)	59 (50.0)	
3–5	25 (42.4)	46 (39.0)	
NA	1 (1.7)	2 (1.7)	
Extranodal involvement			1.00
Yes	25 (42.4)	50 (42.4)	
No	34 (57.6)	68 (57.6)	
Biopsy guided by PET-CT			
Yes	25 (42.4)		
No	34 (57.6)		
Histology of PET-CT guided re-biopsy			
Follicular lymphoma (grade 1-3a)	25 (100)		
SUVmax			
Under 10	31 (52.5)		
10 or over	27 (45.8)		
NA	1 (1.7)		
First-line therapy			
Watch and wait	12 (20.3)	0 (0.0)	
Surgical removal	3 (5.1)	0 (0.0)	
Radiotherapy	10 (17.0)	24 (20.3)	
Immunotherapy	2 (3.4)	1 (0.9)	
Immunochemotherapy	32 (54.2)	82 (69.5)	
Other^a^	0 (0.0)	11 (9.3)	
Relapsed patients	14 (23.7)	73 (61.9)	*< 0.001*
Transformation later in the course of the disease	4 (6.8)	15 (12.7)	0.306

*ECOG *Eastern Cooperative Oncology Group performance status, *FLIPI* Follicular Lymphoma International Prognostic Index, *LDH* lactate dehydrogenase, *NA* not available, *PET-CT* Positron-emission tomography-computed tomography, *SUVmax* Maximum Standardised Uptake Value

^a^Other treatments in the reference group include chlorambucil, COP (cyclophosphamide, vincristine and prednisolone) and CEOP (COP plus epirubicin)

### Prognosis of PET-CT staged patients

**Fig. 2:**
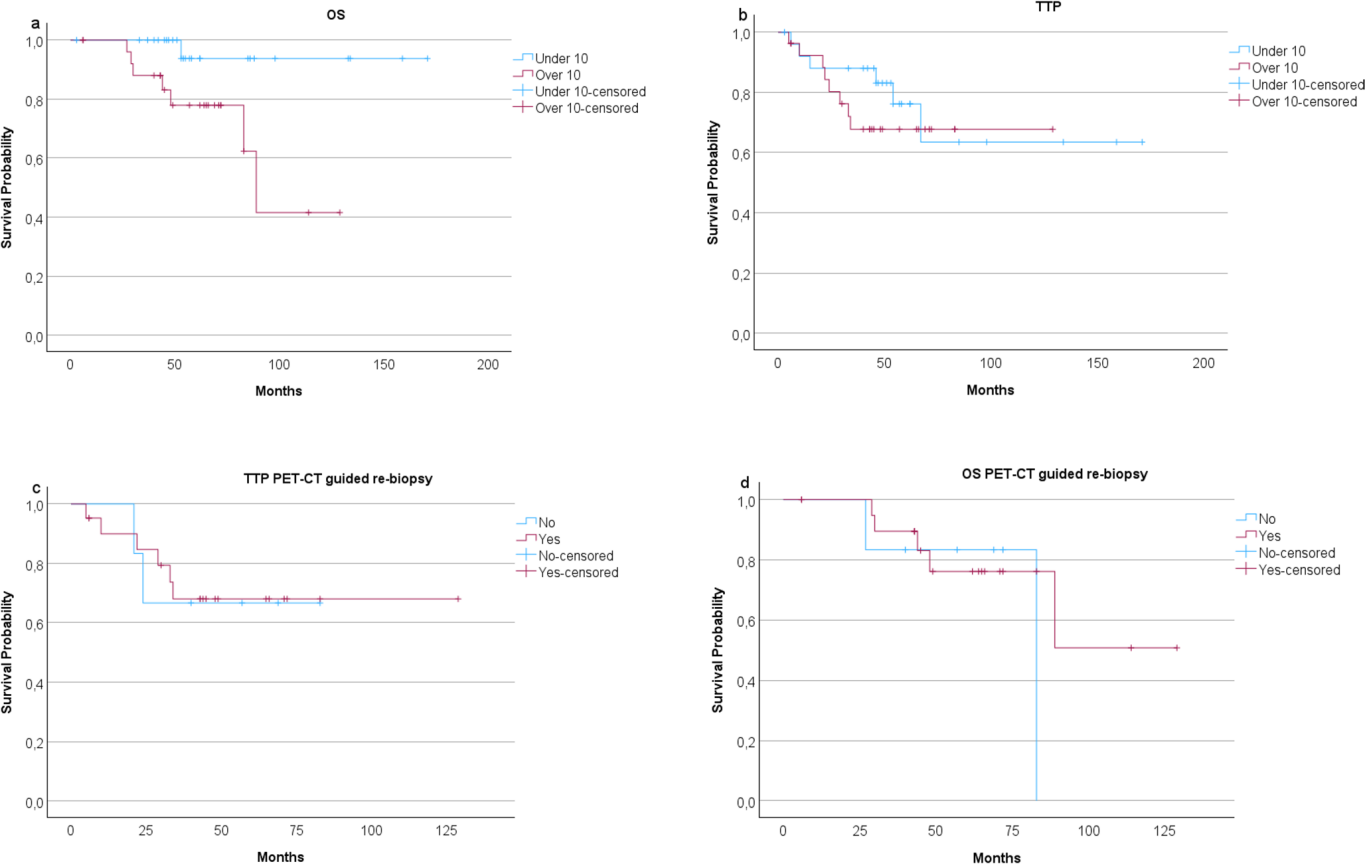
Kaplan-Meier analysis showed (**a**) inferior overall survival (OS) (*p*=0.016) with higher SUVmax values when comparing PET-CT staged patients with follicular lymphoma (grade 1-3a) divided based on reported baseline SUVmax values. **b** Time to progression (TTP) was not significantly different between the groups (*p*=0.488). When (**c**) TTP and (**d**) OS of only patients whose highest SUV values were 10 or more were analysed divided based on whether or not a PET-CT guided biopsy was taken, a re-biopsy was not seen to improve prognosis (*p*=0.904, *p*=0.516)

**Fig. 3:**
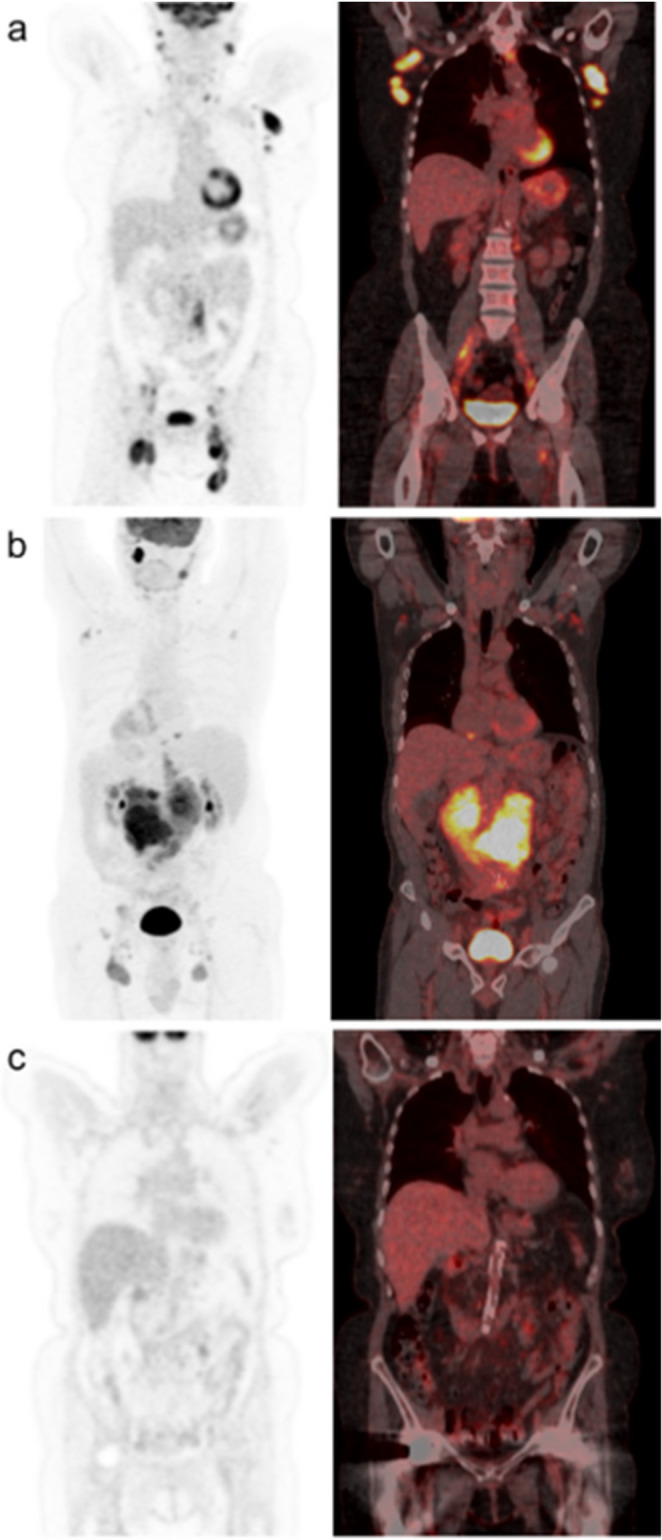
Three patient examples of PET-CT staged patients with follicular lymphoma (FL). **a**, **b** Two patients had a SUVmax of ten or higher at baseline PET-CT and a re-biopsy guided by the results taken from the SUVmax area. The re-biopsies showed no sign of transformation as the histologic finding was grade 2 FL in both examples, and no further transformation was diagnosed in the follow-up. **a** The re-biopsy was taken from the brachium presenting a SUVmax value of 13. **b** The re-biopsy was taken from an area with a SUVmax of 15, which was seen in the abdominal cavity. The patient experienced disease progression within 24 months of started first-line therapy (POD24). **c** The SUVmax of the patient was 8.9 in the parotid gland, and no re-biopsy was performed according to the staging results. Patient soon developed histological transformation to an aggressive non-Hodgkin lymphoma in an unrelated location

In PET-CT staged group, OS was inferior in patients with a high SUVmax (10 or more) compared to those with a SUVmax under 10 (*p* = 0.016), whereas time to progression was not statistically worse with higher SUVmax values (Fig. 2a-b). The median TTP was similar between the group of patients with a reported SUVmax of 10 or higher at 43 months and the group of patients with a SUVmax of under 10 with a median of 52 months (*p* = 0.155). The survival of the patients with high SUVmax (10 or more) did not associate to the fact whether a re-biopsy was taken or not (Fig. 2c-d). A selection of PET-CT staged patient examples is presented in Fig. 3.

### POD24 and relapsed patients

**Fig. 4:**
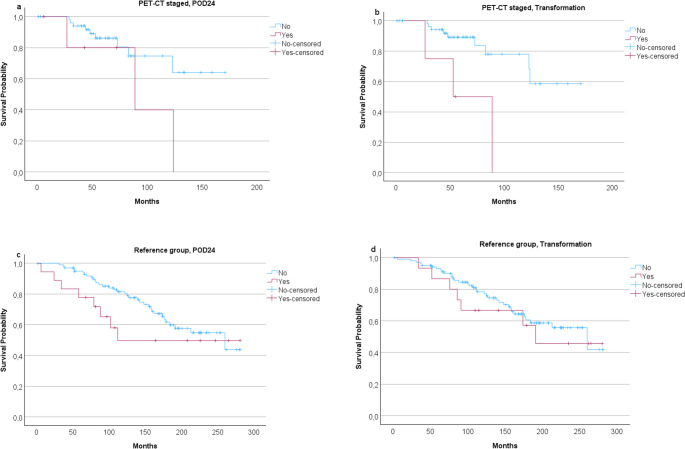
Overall survival (OS) analysis in PET-CT staged subgroup of patients on (**a**) progression of disease in 24 months (POD24) status and (**b**) if the disease transformed to an aggressive B-cell lymphoma or not. A transformed disease might lower OS (*p* = 0.004) whereas POD24 may not (*p* = 0.095). In the reference group neither (**c**) POD24 nor (**d**) transformation was seen to lower OS (*p* = 0.214 and *p* = 0.583, respectively)

 After receiving first-line treatment, six patients (10.2%) in the PET-CT staged group experienced POD24. Correspondingly, in the control patient group, POD24 was observed in 18 patients (15.3%) (*p* = 0.486). However, there was no statistically significant difference noted in OS between POD24 patients and patients whose disease did not progress early in this study population of either PET-CT staged or control patients (*p* = 0.095 and *p* = 0.214, respectively) (Fig. 4a, c). In the study group, the median period from FL diagnosis to first relapse was 21.5 months (range, 5–67). In patients who were not staged with PET-CT, the median period from diagnosis to relapse of disease was 41 months (5-211) (*p* < 0.001). There were significantly more patients with a relapse after initial treatment in the reference group than in the study group.

### Large cell transformed patients

 Among PET-CT staged patients, four (6.8% out of 59 patients) had a histological transformation to an aggressive NHL during the course of the disease. In the control group, 15 (12.7%/118) transformations were diagnosed with histological confirmation during the follow-up (*p* = 0.306). The 5-year cumulative incidence of diagnosed transformations during follow-up was 6.6% in the PET-CT staged group vs. 4.5% in the control group. Treatments of patients at first-line, relapse and transformation are presented in Table 2. The median time from the diagnosis to the confirmed later disease transformation was 37.5 months (range, 6–77) in PET-CT staged patients and in control group 102 months (19–206) (*p* = 0.020). In present data, a transformed disease may predict a worse outcome in the PET-CT staged patients (*p* = 0.004) but not in the reference group (*p* = 0.583) (Fig. 4b, d). The median OS of PET-CT staged patients who demonstrated transformation was seemingly shorter compared to those who did not, 54 vs. 57 months respectively (*p* = 0.507).

**Table 2:** Selected treatment schemes of patients at baseline, following a first relapse and transformation

Variable	PET-CT staged patients *n* (%)	Reference group *n* (%)
Treatment first-line	*n* = 59	*n* = 118
Watch and wait	12 (20.3)	0 (0.0)
Surgical removal	3 (5.1)	0 (0.0)
Radiotherapy	10 (17.0)	24 (20.3)
Immunotherapy	2 (3.4)	1 (0.9)
R-Bendamustine	19 (32.2)	35 (29.7)
R-CHOP	11 (18.6)	17 (14.4)
R-CEOP	2 (3.4)	25 (21.2)
Individually adapted salvage immunochemotherapies	0 (0.0)	5 (4.2)
Other^a^	0 (0.0)	11 (9.3)
First relapse	*n* = 14	*n* = 73
No treatment	0 (0.0)	2 (2.7)
Less intensive therapy^b^	3 (21.4)	19 (26.0)
Salvage immunochemotherapy	7 (50.0)	31 (42.5)
Intensive HDT combined with ASCT	3 (21.4)	18 (24.7)
Other^c^	1 (7.2)	3 (4.1)
Transformed disease	*n* = 4	*n* = 15
Salvage immunochemotherapy	4 (100)	7 (46.7)
Intensive HDT combined with ASCT	0 (0.0)	7 (46.7)
Rituximab	0 (0.0)	1 (6.6)

*ASCT* autologous stem cell transplant, *HDT* high-dose therapy, *R-CEOP* rituximab, cyclophosphamide, epirubicin, vincristine and prednisone, *R-CHOP* rituximab, cyclophosphamide, doxorubicin, vincristine and prednisone

^a^Chlorambucil, chemotherapy with CEOP or COP (cyclophosphamide, vincristine and prednisone)

^b^Included in less intensive therapies are watch and wait, radiotherapy, immunotherapy and O-Mine (obinutuzumab, mesna, ifosfamide, mitoxantrone and etoposide)

^c^Chemotherapy with CEOP, CHOP or MACOP-B (methotrexate, doxorubicin, cyclophosphamide, vincristine, prednisone and bleomycin)

### Prognostic role of PET-CT

**Fig. 5:**
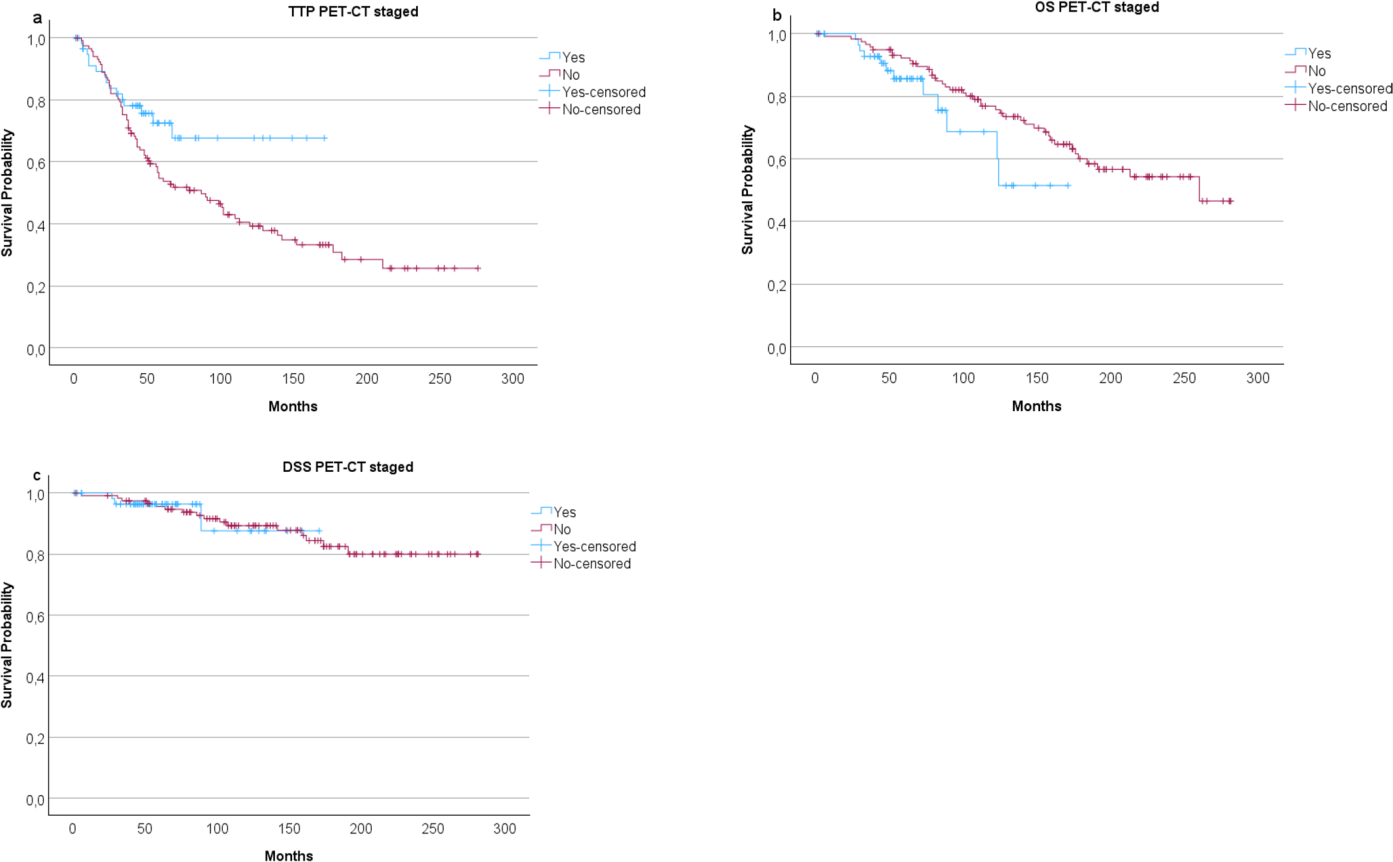
**a** A significant difference was found using Kaplan-Meier survival analysis of time to progression (TTP) comparing groups of patients with grade 1-3a follicular lymphoma staged with or without PET-CT (*p* = 0.038). **b** Overall survival (OS) was not statistically different between the groups (*p* = 0.098). **c** When only lymphoma-related deaths were considered, no significant difference in disease-specific survival (DSS) was noted (*p* = 0.992)

Moreover, we compared the effects of PET-CT on the course of FL between study group and controls. Baseline PET-CT significantly improved time to progression in PET-CT staged patients (*p* = 0.038) (Fig. 5a). In univariate model, PET-CT was the only significant variable (*p* = 0.011) (Table 3). On the contrary, there was no statistical difference found between the groups when OS and DSS were analysed (Fig. 5b-c) (*p* = 0.098 and *p* = 0.992, respectively).

**Table 3:** Unadjusted uni- and multivariate analysis comparing different risk factors of follicular lymphoma and pre-treatment PET-CT in the evaluation of time to progression (TTP) and overall survival (OS)

	HR	95% CI	*p* Value	*p* Value*
TTP				
PET-CT	1.794	1.022–3.148	0.380	0.042
FLIPI	1.200	0.882–1.633	0.365	0.245
Stage	1.120	0.720–1.742	0.575	0.616
SUVmax	1.452	0.502–4.197	0.649	0.491
OS				
PET-CT	0.563	0.283–1.121	0.011	0.102
FLIPI	1.106	0.763–1.602	0.092	0.596
Stage	0.857	0.496–1.482	0.812	0.580
SUVmax	8.561	1.049–69.88	0.390	0.045

*FLIPI *Follicular Lymphoma International Prognostic Index, *HR *hazard ratio, *SUVmax* Maximum Standardised Uptake Value, *95% CI* confidence interval

*p-value of multivariate analysis

## Discussion

This retrospective study of 177 patients of which 59 were staged with diagnostic PET-CT demonstrated that the use of PET-CT and re-biopsy in the case of high SUV values, at the time of the diagnosis, is useful in detecting the patients at the risk of progression of the disease in 24 months. TTP was significantly improved in PET-CT staged patients compared to traditionally staged patients, impeding manifestations of earlier disease progression.

In line with previous findings, our real-world data of PET-CT staged patents suggests that a histological transformation of FL is a factor in predicting poor survival [10], but our cohort size was limited. Improvement in the 10-year OS of patients with FL has been noted in recent years [11, 12] but the heterogeneous clinical course of FL remains as a challenge. High-risk patients are not infallibly detectable with current prognostic indexes. In these cases of clinically unpredictable risks, the published literature supports our findings that baseline PET-CT staging may have a role as a predictive tool. Also, interim evaluation may help to discover those at risk of poor prognosis [13–15]. Therefore, baseline PET-CT imaging and guided re-biopsy may find diseases with primary transformation and thus assist decision-making for first-line treatment with patients diagnosed with FL and delay a progression. In our results TTP was notably longer in patients who were PET-CT staged. The effectiveness of PET in providing results that can have an effect on patient outcomes was also indicated in a retrospective study where PET-staging was seen to decrease the impact of POD24 on prognosis [16].

Pre-treatment PET-CT may prolong further FL progression but not completely rule out POD24 based on our results. Although we did not note a statistically significant difference in OS between POD24 and non-POD24 patients in our study population, POD24 has been recognised as a prognostic factor for inferior OS in previous studies [2–4, 17, 18]. This could be due to the limited number of cases of POD24 in our study population. While the underlying mechanisms of early progression are yet to be specified, transformation has been suggested as an explanator of progression in majority of POD24 patients [19]. In addition, high tumour burden may predict the risk of early progression [20]. Still in common with POD24, no features can fully predict the occurrence of transformation [21]. High FLIPI and LDH levels, B-symptoms, limited response to treatment and rapid changes to a former indolent disease course may precede transformation to an aggressive NHL.

We have previously reported that PET-CT staging and re-biopsy from sites with high SUV value allows for an earlier detection of an otherwise unnoticeable histological transformation [22]. High baseline SUVmax is also associated with the risk of earlier events [23]. According to current literature, a SUVmax of over 10 is generally suspicious of an aggressive disease and therefore indicative of a new biopsy for confirmation of possible transformation [9, 22]. Therefore, high SUVmax is currently used to guide biopsies for confirmation in cases of clinical suspicion of transformation [24–26]. Our findings are consistent with that of a high SUVmax was suggestive of poor survival, even when no re-biopsy was taken, or biopsy was negative for histological transformation. However, in our PET-CT staged cases who underwent transformation after receiving treatment, two patients had a SUVmax of under 10 and two greater than 10, thus initial SUVmax values were not informative of further transformation. Still, from the 34 patients who did not go through a new biopsy after PET-CT there were six patients whose highest SUV exceeded 10 suggesting that the accurate SUVmax cutoff predicting transformation and the need of a re-biopsy remains unclear [22]. Although our results showed no statistical improvement of overall survival in analyses between PET-CT and conventionally staged patients, PET-CT guided re-biopsy seems to be useful in detecting primary transformation, which is essential as an aggressive disease therapying an indication for an anthracycline containing immunochemotherapy. Should patients with histological confirmed FL with high SUVmax values be treated as an aggressive lymphoma, should be further discussed and studied.

As our study was conducted in a retrospective manner, selective bias must be considered when analysing the results. Our PET-CT staged group was made up of patients who were chosen for the scan by clinician based on the individual clinical course of FL. Therefore, the results of PET-CT have had an effect on the treatment planning and possibly the consequent disease course and follow-up of included patients. This also meant that the study group had 12 patients with a watchful waiting approach at first-line. When we repeated our analyses without the patients with a watch and wait approach our results were not affected. The prolonged TTP and smaller number of relapses after initial diagnosis of PET-CT staged patients could be due to the possibility that a more adequate treatment schema could be chosen based on the imaging results. Moreover, the PET-CT group may have been diagnosed at a later date than the controls. There was not a statistically significant difference in OS when comparing the traditionally staged and PET-CT staged groups. This might be because the time of follow-up was seemingly shorter in the PET-CT staged subgroup. Still, in PET-CT staged patients the time between diagnosis and first relapse was shorter than in the controls. Whether the majority of relapses were found because of patients’ symptoms or organized follow-up scans was not significantly different when comparing our subgroups.

Possible inconsistencies in criteria used in the selection of patients for PET-CT are a source of potential bias. From the beginning of the 21 st century the imaging has been started to be utilized for the disease and the use has since been increasing. As we used this to define the study period to start from the beginning of 2000 there were 11 patients in the reference group treated before the rituximab-era. Moreover, some reference patients could have been diagnosed before PET-CT staging was available for use, which increases the risk of bias.

Few studies have researched the effects of PET-CT staging at baseline used for evaluating the risk of POD24 and transformation and the usefulness of PET-CT guided re-biopsy in patients with grade 1-3a FL [16, 22]. The precise data collection and inclusion of only patients with low-grade FL ensure the relative coherence of the material. However, this is a retrospective study including a limited population from a single center.

Based on our data, PET-CT and potential re-biopsy of lesions with high SUV values at diagnosis is a useful risk assessment tool in FL. POD24 remains unforeseeable, and it still is deemed to be a significant prognostic factor requiring more accurate identifying practices. Considering the indolent characteristics of FL, research with longer follow-up periods and large-scale study populations is needed to validate our conclusions. Efforts in further studies should focus on ways to discover patients at-risk for progression and those with transformed disease at the earliest stage possible for optimal therapeutical decision making and patient outcomes.

## Data Availability

No datasets were generated or analysed during the current study.
